# Supplementary material for: Implementing the Point Spread Function Deconvolution for Better Molecular Characterization of Newly Diagnosed Gliomas: A Dynamic 18F-FDOPA PET Radiomics Study
Source: Cancers (Basel). 2022 Nov 23;14(23):5765. doi: 10.3390/cancers14235765 (PMC9738921; doi:10.3390/cancers14235765)
Supplement: Supplementary file 1 [file cancers-14-05765-s001.zip › Supplementary.pdf]

Supplementary Table S1. Percent of extracted features changed by applying the PSFd.

<table>
<tr><th colspan="3">Voxel-Based Analysis</th></tr>
<tr><th>Feature Name</th><th>Percent of Static TBR Radiomics Features Changed *</th><th>Percent of Dynamic TTP Radiomics Features Changed</th></tr>
<tr><td>Morphology Volume (mesh-based)</td><td colspan="2">0.15%</td></tr>
<tr><td>Morphology Volume (counting) / MTV</td><td colspan="2">0.16%</td></tr>
<tr><td>Morphology Surface area</td><td colspan="2">5.20%</td></tr>
<tr><td>Morphology Surface to volume</td><td colspan="2">−4.78%</td></tr>
<tr><td>Morphology Sphericity</td><td colspan="2">−2.04%</td></tr>
<tr><td>Morphology Maximum 3D diameter</td><td colspan="2">3.13%</td></tr>
<tr><td>Morphology Major axis length</td><td colspan="2">4.01%</td></tr>
<tr><td>Morphology Minor axis length</td><td colspan="2">2.65%</td></tr>
<tr><td>Morphology Least axis length</td><td colspan="2">1.63%</td></tr>
<tr><td>Morphology Elongation</td><td colspan="2">−1.12%</td></tr>
<tr><td>Morphology Flatness</td><td colspan="2">−1.78%</td></tr>
<tr><td>Local intensity Local intensity peak</td><td>7.20%</td><td>−3.99%</td></tr>
<tr><td>Local intensity Global intensity peak</td><td>8.43%</td><td>9.53%</td></tr>
<tr><td>Statistics Mean / TBR_{mean}</td><td>3.74%</td><td>2.61%</td></tr>
<tr><td>Statistics Variance</td><td>38.77%</td><td>6.41%</td></tr>
<tr><td>Statistics Skewness</td><td>11.04%</td><td>2.36%</td></tr>
<tr><td>Statistics (Excess) kurtosis</td><td>69.65%</td><td>34.37%</td></tr>
<tr><td>Statistics Median</td><td>2.61%</td><td>2.44%</td></tr>
<tr><td>Statistics Minimum</td><td>0.00%</td><td>1.26%</td></tr>
<tr><td>Statistics 10th percentile</td><td>0.45%</td><td>−0.25%</td></tr>
<tr><td>Statistics 90th percentile</td><td>7.09%</td><td>4.03%</td></tr>
<tr><td>Statistics Maximum / TBR_{max}</td><td>13.20%</td><td>1.67%</td></tr>
<tr><td>Statistics Interquartile range</td><td>15.82%</td><td>5.75%</td></tr>
<tr><td>Statistics Range</td><td>23.95%</td><td>1.67%</td></tr>
<tr><td>Statistics Mean absolute deviation</td><td>18.53%</td><td>4.81%</td></tr>
<tr><td>Statistics Robust mean absolute deviation</td><td>16.57%</td><td>5.87%</td></tr>
<tr><td>Statistics Energy</td><td>7.46%</td><td>0.44%</td></tr>
<tr><td>Statistics Root mean square</td><td>4.38%</td><td>2.90%</td></tr>
<tr><td>Intensity histogram Entropy</td><td>8.04%</td><td>2.33%</td></tr>
<tr><td>Intensity histogram Uniformity</td><td>−19.56%</td><td>−9.05%</td></tr>
<tr><td>GLCM Joint maximum</td><td>−27.13%</td><td>−6.61%</td></tr>
<tr><td>GLCM Joint average</td><td>3.94%</td><td>2.41%</td></tr>
<tr><td>GLCM Joint variance</td><td>39.74%</td><td>6.60%</td></tr>
<tr><td>GLCM Joint entropy</td><td>9.30%</td><td>2.43%</td></tr>
<tr><td>GLCM Difference average</td><td>22.37%</td><td>2.82%</td></tr>
<tr><td>GLCM Difference variance</td><td>45.73%</td><td>3.72%</td></tr>
<tr><td>GLCM Difference entropy</td><td>11.66%</td><td>2.03%</td></tr>
<tr><td>GLCM Sum average</td><td>3.94%</td><td>2.41%</td></tr>
<tr><td>GLCM Sum entropy</td><td>6.84%</td><td>2.04%</td></tr>
<tr><td>GLCM Angular second moment</td><td>−36.95%</td><td>−14.00%</td></tr>
<tr><td>GLCM Contrast</td><td>47.06%</td><td>3.26%</td></tr>
<tr><td>GLCM Dissimilarity</td><td>22.37%</td><td>2.82%</td></tr>
<tr><td>GLCM Inverse difference</td><td>−7.14%</td><td>−1.91%</td></tr>
<tr><td>GLCM Inverse difference normalized</td><td>−0.29%</td><td>−0.08%</td></tr>
<tr><td>GLCM Inverse difference moment</td><td>−9.09%</td><td>−2.52%</td></tr>
</table>

GLCM Inverse difference moment normalized	−0.03%	0.01%
GLCM Inverse variance	−1.53%	−0.61%
GLCM Correlation	1.80%	1.24%
GLCM Autocorrelation	9.58%	3.02%
GLCM Cluster tendency	39.49%	6.87%
GLCM Cluster shade	91.09%	6.74%
GLCM Cluster prominence	95.99%	7.01%
GLCM Information correlation 1	−4.43%	−0.54%
GLCM Information correlation 2	1.77%	0.61%
GLRLM Short runs emphasis	4.55%	0.87%
GLRLM Long runs emphasis	−15.21%	−7.50%
GLRLM Low grey level run emphasis	−4.58%	−8.48%
GLRLM High grey level run emphasis	9.52%	3.37%
GLRLM Short run low grey level emphasis	0.01%	−8.31%
GLRLM Short run high grey level emphasis	14.18%	3.88%
GLRLM Long run low grey level emphasis	−18.70%	−12.87%
GLRLM Long run high grey level emphasis	−8.18%	−2.23%
GLRLM Grey level non-uniformity	−4.02%	−3.53%
GLRLM Grey level non-uniformity normalized	−20.47%	−9.11%
GLRLM Run length non-uniformity	11.99%	3.67%
GLRLM Run length non-uniformity normalized	8.85%	1.55%
GLRLM Run percentage	5.98%	1.27%
GLRLM Grey level variance	40.08%	6.04%
GLRLM Run length variance	−20.40%	−10.53%
GLRLM Run entropy	2.72%	1.04%
GLSZM Small zone emphasis	18.39%	1.20%
GLSZM Large zone emphasis	−45.90%	−28.28%
GLSZM Low grey level emphasis	−6.36%	−21.90%
GLSZM High grey level emphasis	12.45%	3.75%
GLSZM Small zone low grey level emphasis	12.23%	−13.91%
GLSZM Small zone high grey level emphasis	32.40%	4.09%
GLSZM Large zone low grey level emphasis	−46.21%	−32.41%
GLSZM Large zone high grey level emphasis	−45.50%	−16.94%
GLSZM Grey level non-uniformity	48.24%	4.97%
GLSZM Grey level non-uniformity normalized	−23.04%	−15.53%
GLSZM Zone size non-uniformity	68.87%	6.75%
GLSZM Zone size non-uniformity normalized	8.71%	0.69%
GLSZM Zone percentage	74.10%	1.32%
GLSZM Grey level variance	40.57%	3.46%
GLSZM Zone size variance	−44.36%	−27.96%
GLSZM Zone size entropy	8.92%	1.45%
NGTDM Coarseness	−32.83%	−22.34%
NGTDM Contrast	10.14%	3.22%
NGTDM Busyness	−18.69%	−1.77%
NGTDM Complexity	78.05%	2.56%
NGTDM Strength	70.32%	−9.62%
NGLDM Low dependence emphasis	40.03%	1.90%
NGLDM High dependence emphasis	−20.68%	−5.11%
NGLDM Low grey level count emphasis	−4.34%	−6.74%
NGLDM High grey level count emphasis	9.09%	3.11%

NGLDM Low dependence low grey level emphasis	30.35%	−8.46%
NGLDM Low dependence high grey level emphasis	59.59%	5.49%
NGLDM High dependence low grey level emphasis	−22.80%	−7.32%
NGLDM High dependence high grey level emphasis	−17.01%	−1.65%
NGLDM Grey level non-uniformity	−9.20%	−6.84%
NGLDM Dependence count non-uniformity	8.19%	4.01%
NGLDM Dependence count non-uniformity normalized	9.28%	0.09%
NGLDM Grey level variance	38.91%	6.42%
NGLDM Dependence count variance	−11.01%	−0.68%
NGLDM Dependence count entropy	2.11%	1.35%
**Region-based analysis**		
**Feature name**	**Percent of static TBR conventional features changed**	**Percent of dynamic features changed**
TBR_{peak}	8.43%	–
TSR_{mean}	−1.52%	–
TSR_{max}	7.56%	–
TSR_{peak}	3.00%	–
TTP	–	9.29%
Slope	–	14.26%

Values in red indicate significant changes (p-value<0.05 for features compared before and after applying the PSFd). * Common radiomics features for voxel/region-based analysis. PSFd: point spread function deconvolution; TBR: tumor-to-background-ratio; TSR: tumor-to-striatum-ratio; TTP: time-to-peak; GLCM: grey level co-occurrence matrix; GLRLM: grey level run length matrix; GLSZM: grey level size zone matrix; NGTDM: neighborhood grey tone difference matrix; NGLDM: neighboring grey level dependence matrix.

Supplementary Table S2. Model performance on the train set for IDH-mutation prediction.

	**Without PSFd**				**With PSFd**			
**Features / Metrics**	**AUC**	**Sensitivity**	**Specificity**	**B_ACC**	**AUC**	**Sensitivity**	**Specificity**	**B_ACC**
**Voxel-based analysis**								
**Static**	0.820 [0.812, 0.829]	0.863 [0.852, 0.874]	0.595 [0.580, 0.610]	0.729 [0.719, 0.739]	0.825 [0.818, 0.832]	0.895 [0.886, 0.904]	0.565 [0.554, 0.577]	0.730 [0.723, 0.738]
**Dynamic**	0.840 [0.834, 0.847]	0.799 [0.789, 0.809]	0.687 [0.676, 0.698]	0.743 [0.736, 0.751]	0.835 [0.828, 0.842]	0.792 [0.780, 0.804]	0.697 [0.687, 0.707]	0.744 [0.737, 0.752]
**Static/ Dynamic**	0.905 [0.899, 0.911]	0.899 [0.888, 0.910]	0.737 [0.727, 0.747]	0.818 [0.809, 0.827]	0.897 [0.892, 0.902]	0.875 [0.866, 0.883]	0.732 [0.722, 0.742]	0.803 [0.797, 0.810]
**Region-based analysis**								
**Static/ Dynamic**	0.903 [0.899, 0.908]	0.812 [0.793, 0.829]	0.845 [0.835, 0.855]	0.828 [0.821, 0.836]	0.916 [0.912, 0.920]	0.746 [0.730, 0.763]	0.897 [0.891, 0.903]	0.821 [0.814, 0.829]

Results are presented as a mean followed by the 95% CI in brackets. AUC: areas under the curve; B_ACC: balanced accuracy; PSFd: point spread function deconvolution.

Supplementary Table S3. Model performance on the train set for 1p/19q codeletion prediction.

	Without PSFd				With PSFd			
**Features / Metrics**	**AUC**	**Sensitivity**	**Specificity**	**B_ACC**	**AUC**	**Sensitivity**	**Specificity**	**B_ACC**
**Voxel-based analysis**								
**Static**	0.885 [0.879, 0.892]	0.935 [0.921, 0.949]	0.715 [0.705, 0.726]	0.825 [0.816, 0.834]	0.884 [0.878, 0.890]	0.904 [0.885, 0.921]	0.729 [0.719, 0.739]	0.816 [0.807, 0.825]
**Dynamic**	0.940 [0.935, 0.945]	0.910 [0.896, 0.924]	0.816 [0.808, 0.825]	0.863 [0.854, 0.872]	0.920 [0.915, 0.925]	0.876 [0.860, 0.891]	0.795 [0.785, 0.805]	0.835 [0.825, 0.845]
**Static/ Dynamic**	0.962 [0.958, 0.966]	0.941 [0.931, 0.952]	0.863 [0.854, 0.872]	0.902 [0.895, 0.909]	0.968 [0.966, 0.971]	0.944 [0.932, 0.956]	0.852 [0.845, 0.859]	0.898 [0.892, 0.905]
**Region-based analysis**								
**Static/ Dynamic**	0.959 [0.955, 0.963]	0.893 [0.879, 0.907]	0.844 [0.836, 0.853]	0.869 [0.862, 0.876]	0.962 [0.958, 0.967]	0.900 [0.888, 0.912]	0.888 [0.881, 0.895]	0.894 [0.887, 0.901]

Results are presented as a mean followed by the 95% CI in brackets. AUC: areas under the curve; B_ACC: balanced accuracy; PSFd: point spread function deconvolution.
